# The experiences of patients with advanced heart failure, family carers, and health professionals with palliative care services: a secondary reflexive thematic analysis of longitudinal interview data

**DOI:** 10.1186/s12904-023-01241-1

**Published:** 2023-08-10

**Authors:** Bader Nael Remawi, Amy Gadoud, Nancy Preston

**Affiliations:** 1 Lancaster Medical School, Lancaster University, Lancaster, LA1 4AT UK; 2 Doctor of Pharmacy Department, Birzeit University, Birzeit, Palestine; 3 Lancaster Medical School, Lancaster University, Lancaster, LA1 4AT UK; 4 Division of Health Research, Lancaster University, Lancaster, LA1 4AT UK

**Keywords:** Palliative care, Heart failure, Needs assessment, Qualitative research, Normalisation process theory, Reflexive thematic analysis, Secondary data analysis

## Abstract

**Background:**

Patients with heart failure have significant palliative care needs, but few are offered palliative care. Understanding the experiences of delivering and receiving palliative care from different perspectives can provide insight into the mechanisms of successful palliative care integration. There is limited research that explores multi-perspective and longitudinal experiences with palliative care provision. This study aimed to explore the longitudinal experiences of patients with heart failure, family carers, and health professionals with palliative care services.

**Methods:**

A secondary analysis of 20 qualitative three-month apart interviews with patients with heart failure and family carers recruited from three community palliative care services in the UK. In addition, four group interviews with health professionals from four different services were analysed. Data were analysed using ‘reflexive thematic’ analysis. Results were explored through the lens of Normalisation Process Theory.

**Results:**

Four themes were generated: Impact of heart failure, Coping and support, Recognising palliative phase, and Coordination of care. The impact of heart failure on patients and families was evident in several dimensions: physical, psychological, social, and financial. Patients developed different coping strategies and received most support from their families. Although health professionals endeavoured to support the patients and families, this was sometimes lacking. Health professionals found it difficult to recognise the palliative phase and when to initiate palliative care conversations. In turn, patients and family carers asked for better communication, collaboration, and care coordination along the whole disease trajectory.

**Conclusions:**

The study provided broad insight into the experiences of patients, family carers, and health professionals with palliative care. It showed the impact of heart failure on patients and their families, how they cope, and how they could be supported to address their palliative care needs. The study findings can help researchers and healthcare professionals to design palliative care interventions focusing on the perceived care needs of patients and families.

**Supplementary Information:**

The online version contains supplementary material available at 10.1186/s12904-023-01241-1.

## Background

Heart failure is a complex, progressive clinical syndrome that affects approximately 38 million people worldwide [1]. It causes frequent hospitalisations and has a higher mortality risk than common types of cancer [1–3]. Recent advancements in heart failure therapy have raised the mean life expectancy, meaning patients may live longer with their advanced disease [4]. Patients with heart failure experience breathlessness, fatigue, and other symptoms which are comparable to those of cancer [5, 6]. The caregiving role provided by family carers can be burdensome, physically demanding, and emotionally difficult [7, 8].

Given the progressive nature and significant burden of heart failure on patients and their families, the goals of care shift towards improved quality of life [9], where a palliative care approach plays an important role [10]. Palliative care is a team-based approach and is typically classified into generalist palliative care (provided by the usual care team) and specialist palliative care (provided by a multidisciplinary team with specialist palliative care training) [11, 12]. Many palliative care needs of patients with heart failure could be addressed by palliative care generalists [13]. Referral to specialist palliative care is reserved for those with severe and intractable problems [12].

Providing palliative care for patients with heart failure improves quality of life, symptoms, functional status, mental health, and satisfaction with care, without affecting survival [14–19]. Palliative care involvement reduces family carers’ caregiving burden, improves their mental health, and reduces medical service utilisation and healthcare costs [7, 14, 17–20]. Given these benefits, multiple international guidelines have called for integrating palliative care into standard heart failure care throughout the whole illness trajectory [4, 21–24]. Despite this, most patients with heart failure have poor or late access to palliative care compared to those with cancer [25–27].

Qualitative studies have been conducted to explore the palliative care needs of patients with heart failure and family carers [28, 29]. However, most studies were of cross-sectional design, providing a snapshot of palliative care needs rather than reflecting changes over the illness trajectory [30]. Studies about the experiences of patients and family carers with palliative care services are lacking, as most studies focused on the perspectives of healthcare professionals [31]. Understanding the experiences of delivering and receiving palliative care from different perspectives can provide insight into the mechanisms of successful palliative care integration [32], and subsequently develop effective interventions that would improve patient care [31, 33].

This study aimed to gain a greater understanding of the experiences of patients with heart failure receiving palliative care services, their family carers, and health professionals involved in the patient’s care, to develop a future needs-based palliative care intervention. Specific objectives were:

- Evaluate the perceptions of patients with heart failure receiving palliative care, their family carers, and health professionals involved in the patient’s care on the holistic palliative care needs of the patients and families.
- Identify the key health professionals involved in the patient’s care who address these needs.
- Explore how the health professionals involved in the patient’s care address these needs.

## Methods

### Design

A secondary analysis of qualitative interview data was conducted. Interviews were with service providers and users who were delivering and experiencing palliative care, respectively. Findings were explored using Normalisation Process Theory [34, 35]. Normalisation Process Theory is built up around four constructs that explain the mechanisms of the routine incorporation of an organisational practice into everyday life [34, 35] (Table 1). It was used as a theoretical framework to trigger thinking about the issue of integrating and implementing palliative care in clinical practice [36].

**Table 1:** Normalisation process theory constructs

Normalisation process theory constructs	Definitions
Coherence (sense-making)	Assessing the components of the new intervention; including its relationship with current practices and potential value to the users
Cognitive participation (relation-building)	Assessing the people involved in the new intervention; including engaging and enrolling people to join in and support the intervention
Collective action (operationalisation)	Assessing the enacting of the new intervention; including allocating resources and doing tasks to support the intervention
Reflexive monitoring (appraisal)	Assessing the outcomes of the new intervention; including giving feedback on the intervention’s effects on users and those around them

### Summary of the primary study

The primary study, which provided the data for the secondary analysis, was a part of the EU-funded research project Integrated Palliative Care in Cancer and Other Chronic Conditions (InSup-C) which aimed to understand the mechanisms of successful palliative care integration across Europe [32]. The primary study aimed to investigate the experiences of patients with advanced heart failure, Chronic Obstructive Pulmonary Disease (COPD), and cancer; family carers; and health professionals with palliative care provision. It was conducted in 23 palliative care services across five European countries over five years (2012–2016), including five services in the UK.

Details of the inclusion criteria and recruitment process of patients, family carers, and health professionals are available in the primary study protocol paper [32]. Semi-structured individual interviews were conducted with patients and family carers at baseline and after three months. If the patient died during this time, family carers were invited to a follow-up *bereavement* interview. Semi-structured group interviews were conducted with health professionals at the end of data collection. In the UK, 35 patients and 13 family carers participated in individual interviews, while 23 health professionals participated in four group interviews. The interview topic guides for the patient, family carer, and health professional interviews were published previously [37–39] and are collated in Additional file 1.

The findings of the primary study showed the unmet palliative care needs of patients and family carers and the importance of ongoing multidisciplinary care [37–40]. These findings were not specific to patients with heart failure, but rather to the whole cohort of patients. The data related to heart failure had not been analysed separately and therefore they were subject to further, more specific, analysis.

### Data collection

Only UK data relevant to patients with heart failure were included in the secondary data analysis. The data included transcripts of patient and family carer interviews, transcripts of health professional group interviews, and demographic characteristics of interviewed participants. There were 20 individual interviews with patients with heart failure and family carers recruited from three community palliative care services, and four group interviews with health professionals from four different services (Table 2). Although this study is a secondary data analysis where determining the sample size was beyond our control, these numbers of interviews agree with the minimum recommendations of more than ten interviews for relatively broad-scope studies that address sensitive topics in heterogeneous participants [41].

**Table 2:** Number of patients with heart failure, family carers, and health professionals interviewed in the UK

Individual interviews	Baseline		Month-3		
Patients	7		5 (2 patients died)		
Family carers	5		5 (including 2 bereavement interviews)		
Total	11 (one joint interview)		9 (one joint interview)		
Group interviews	**Group interview-1**	**Group interview-2**		**Group interview-3**	**Group interview-4**
Health professionals	6	6		5	6

### Data analysis

The interviews were analysed following Braun and Clarke’s approach to reflexive thematic analysis, which endorses the active involvement of the researcher in the analysis process [42, 43]. The main coding approach was inductive (developing themes from the data content) and semantic (staying close to participants’ terms and presenting a descriptive account of their experiences with palliative care) [42]. Still, coding went sometimes beyond the surface meanings of the data and presented a more conceptual account of the underpinning assumptions (latent) [42]. The themes were mapped onto the Normalisation Process Theory constructs which triggered thinking about implementing palliative care into healthcare practice [44].

The analysis was conducted by BR who was not involved in the original study, with regular feedback from NP (one of the primary researchers) and AG. It was aided by NVivo-12 Plus qualitative data analysis software and reported using the Best Practice for Reporting Reflexive Thematic Analysis guidance [41]. The six phases for reflexive thematic analysis were followed iteratively [42, 45]. This involved familiarising with the data through reading and re-reading the interviews’ transcripts and writing analytic observations, creating codes across the dataset and collating relevant data extracts, clustering the codes to create potential subthemes and candidate themes, checking the candidate themes against the coded extracts and all interviews, naming and describing the themes and linking them in a thematic map, and finally presenting the themes with supporting data extracts in the report. The validity of the analysis findings was checked using Braun and Clarke’s Checklist of Criteria for Good Thematic Analysis [42] (Additional file 2).

### Ethical considerations

The primary study was approved by the relevant research ethics committees [32], and approval from the chief investigator was gained to use the data for secondary analysis. Ethics approval for the secondary data analysis was obtained from the Faculty of Health and Medicine Research Ethics Committee at Lancaster University on 26th May 2020 (reference number: FHMREC19099).

## Results

### Participants

The demographic characteristics of the interviewed patients and family carers are displayed in Table 3. The mean age of patients and family carers was 70 and 71 years, respectively. One patient-family carer dyad was interviewed jointly both at baseline and follow-up. Two patients did not have a family carer and two other patients died before their follow-up interview while their relatives took part in a bereavement interview. The demographic characteristics of the interviewed health professionals are displayed in Table 4. Nurses were the majority in all group interviews.

**Table 3:** Demographic characteristics of interviewed patients and family carers

	Age range (years)	Relationship	Follow-up interview	Joint interview
Patient-1	70–79	Spouse	Died	No
Carer-1	70–79		Yes (bereavement)	
Patient-2	70–79	Spouse	Died	No
Carer-2	60–69		Yes (bereavement)	
Patient-3	50–59	Spouse	Yes	No
Carer-3	50–59		Yes	
Patient-4	70–79	no family carer	Yes	--
Patient-5	80–89	Spouse	Yes	Yes
Carer-5	90–99		Yes	
Patient-6	80–89	no family carer	Yes	--
Patient-7	40–49	Mother	Yes	No
Carer-7	70–79		Yes	

**Table 4:** Demographic characteristics of interviewed health professionals

Group interviews	Interview-1	Interview-2	Interview-3	Interview-4
Participants, n	6	6	5	6
Age (years), mean	48	48	44	51
Female gender, n	6	6	5	4
Profession, n				
Palliative medicine consultant	1	0	0	1
General practitioner	1	1	0	1
Nurse	3	4	4	3
Physiotherapist	0	0	1	0
Occupational therapist	0	0	0	1
Social worker	0	1	0	0
Chaplain	1	0	0	0
Years of qualification, mean	22	20	16	25
Years in post, mean	6	6	5	9

### Themes, subthemes, and thematic map

Codes were created from both the patient and family carer interviews and the health professional group interviews. These codes were collated into subthemes, which were further clustered together to generate four major themes of the experiences of patients, family carers, and health professionals with palliative care services (Table 5). The themes were shared across the patient, family carer, and health professional interviews. Theme-1 (Impact of heart failure) and Theme-2 (Coping and support) were more prominent in the patient and family carer interviews, while Theme-3 (Recognising palliative phase) and Theme-4 (Coordination of care) were more prominent in the health professional interviews.

**Table 5:** Themes and subthemes of the experiences with palliative care services

Themes	Subthemes
Theme-1: Impact of heart failure	Impact of heart failure on patients
	Impact of heart failure on families
Theme-2: Coping and support	Patient coping
	Patient support
Theme-3: Recognising palliative phase	Identifying palliative patients
	Palliative care conversations
Theme-4: Coordination of care	Networking
	Continuity of care

**Fig. 1:**
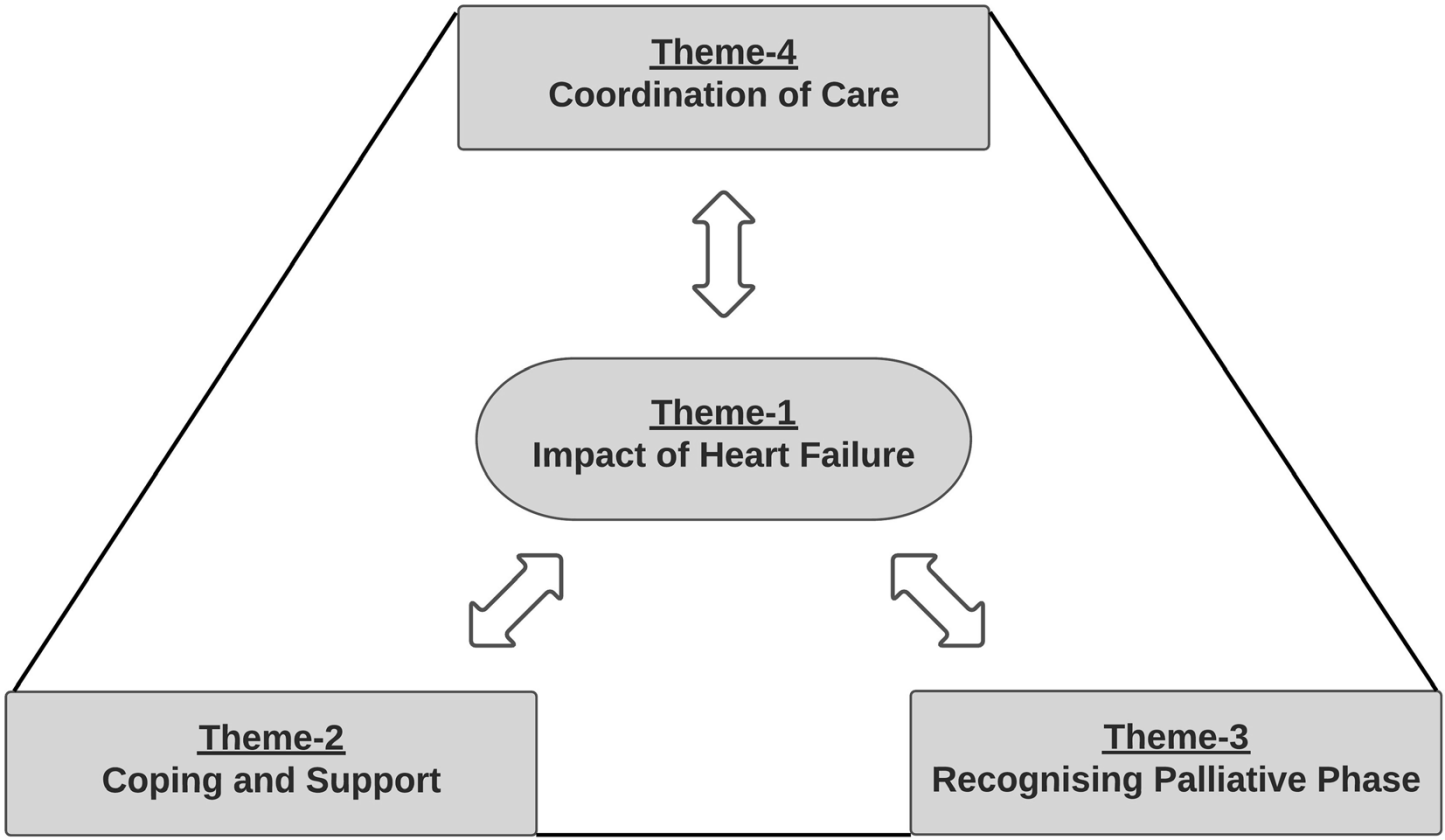
Thematic map of the experiences with palliative care services

The relationship between the themes is represented in a thematic map (Fig. 1). To relieve the impact of heart failure on patient and family lives, the need for coping and support, recognising the palliative phase, and coordination of care became evident. Once the palliative phase is recognised, patients and families can be provided with coordinated care and professional support. In turn, care coordination is needed for optimal patient and family support and for timely palliative care conversations.

The four inductively developed themes were mapped onto the Normalisation Process Theory constructs (Table 6). In response to the multidimensional impact of heart failure on patients and families (Theme-1), the value of identifying and addressing their palliative care needs became evident to relieve this impact (Coherence). As a result, patients developed coping strategies with the help of families, while health professionals endeavoured to provide support (Theme-2) and operationalise palliative care to address the unmet needs (Collective action). Health professionals needed to identify patients with palliative needs and engage in palliative care conversations (Theme-3); they agreed that this should be part of their work despite the expected challenges (Cognitive participation). Patients, family carers, and health professionals commented on the quality of communication and care coordination (Theme-4); they assessed and provided feedback on the palliative care services and pointed to areas where further support is needed (Reflexive monitoring).

**Table 6:** Mapping the themes onto Normalisation Process Theory constructs

Normalisation process theory constructs	Corresponding themes
Coherence (sense-making work, meaning)	Theme-1: Impact of heart failure
Collective action (operational work, effort)	Theme-2: Coping and support
Cognitive participation (relational work, commitment)	Theme-3: Recognising palliative phase
Reflexive monitoring (appraisal work, feedback)	Theme-4: Coordination of care

### Theme-1: impact of heart failure

This theme reflects the significant impact of heart failure on the daily lives of patients and families and the needs that emerged from this impact. Heart failure affected patients and families in several dimensions: physical, psychological, social, and financial, while the spiritual impact was less evident in the interviews. Patients also had limitations in activities of daily living and information needs.

#### Impact of heart failure on patients

Patients experienced ongoing physical symptoms such as general weakness, limited mobility, leg swelling, breathlessness, and pain which made their lives difficult. As a result, patients were limited in their activities of daily living and became dependent on carers:

(Patient-5_Joint interview_Baseline): There was a bath at that end of the bathroom. Well, we never take a bath. Who wants a bath at this age? We’re all frightfully good at getting in it but [chuckling] we can’t get out.

Patients’ ability to go out and drive was restricted; making them housebound and isolated. Most had to leave their jobs which affected their wellbeing and sense of self and caused financial struggle. Patients experienced comorbidities such as cancer and kidney failure which further exacerbated the physical symptoms of heart failure.

Patients experienced significant mental health issues. They were afraid of the unknown and had feelings of uncertainty and insecurity as they were facing an ambiguous future. This uncertainty prevented them from planning effectively for the present and future:

(Patient-2_Baseline): it’s the whole thing of not knowing what’s going to happen when you wake up in a morning, how the day’s going to be. We can’t book anything because you just don’t know… what the future’s going to be.

Patients experienced poor self-confidence as their role changed from an independent to a dependent person and were worried about being a burden on their families. The need for mental health support, compassion, and empathy from family and health professionals was evident. Some patients described spiritual issues such as hopelessness, isolation, and withdrawal from life, and others experienced depression because of physical suffering:

(Patient-3_Baseline): …because I sometimes get really bad days where, despite taking all my painkillers and doing all the right things, I’m in extreme agony and I can’t get out of bed or I can’t get dressed or, you know, I just go into kind of a depression because I’m just in too much pain, I just want to be left alone.

Heart failure left the patients with several questions to ask about their condition. Patients wanted up-to-date information about what is happening with their heart in clear and simple language. Some did not know what causes their symptoms and attributed them to ageing or worrying. Patients also needed information on self-care and medications. They were not fully aware of the available care services and their role, including what palliative care and hospices could offer, and whether they were eligible for hospice care. They wanted to know whether, when, and whom to call for professional help when they have symptoms:

*“(Palliative medicine consultant): I think there’s another potential problem – not particular to palliative care – but for people with long-term conditions… of services tending to become only reactive to them […] because a lot of patients and [family] carers don’t know how bad a problem needs to get to justify calling someone…” (Group interview-4).*

#### Impact of heart failure on families

Caring for patients with heart failure had a physical, psychological, social, and financial impact on families. Because of ongoing caring responsibilities, some family carers had to give up work to care for their relative. Families were worried about the patient’s condition and not being able to cope with their care needs anymore. Some felt they were living in a separate world as they employed all their time caring for their relative:

(Family carer-1_Bereavement): I was in a different world really. I’d been caring for him for so long that I think my… my own self had kind of gone into the background […] and I’m, you know, I’m under 8 stone now, which is… I’ve lost a lot of weight because I suppose I haven’t been looking after myself at all. I mean I’m not aware that I’m not looking after myself but obviously it’s taken its toll...

Heart failure disrupted the relationship between patients and family carers. The family role changed to a caring role to help patients cope with their illness, while patients were busy dealing with their illness and did not have time to look after their family carers.

Family carers were not fully aware of the available care services and their role. They did not know if patients with heart failure were eligible for palliative care. Families were uncertain about the role and remit of some healthcare professionals and had unrealistic expectations that conflicted with what care services could offer:

*“(Palliative care clinical nurse specialist): I think, going back to what makes good integrated palliative care for patients, again it is about services but it’s also about making… ensuring that the family know what services are available and what their expectations can be, because it can be just as equally as detrimental by misinforming people about services and then you can’t deliver. And certainly there have been a couple of instances where things have broken down because families’ expectations have exceeded way over what could possibly be given.” (Group interview-3).*

### Theme-2: coping and support

This theme describes the coping process of patients and the professional and family support provided to relieve the impact of heart failure on their lives. Patients developed different coping strategies in response to the heart failure impact although some were struggling to cope effectively. Health professionals endeavoured to support the patients in their disease journey, but the outcomes were not always perceived as successful. Because of patients’ diverse care needs, multiple health professionals were involved. Families had a key role in providing most of the patient care but some struggled and felt overburdened.

#### Patient coping

Patients acknowledged and accepted the reality of being ill with heart failure and recognised the progressive, life-limiting nature of their illness. They had to live with their limitations and were convinced that nothing more could be done to eliminate their symptoms. As patients accepted the reality of heart failure, they adapted and adjusted to their limitations by doing their daily activities at a slow pace and in small stages:

(Patient-2_Baseline): The flesh is weak, yes. The brain says you can do it and, like a lot of things, and I’ve learnt you can do mammoth tasks if you take it in tiny bits.

Patients tried to cope with and work through their difficulties. Some patients coped by setting themselves attainable goals and pushing themselves to achieve them. Patients strived to keep independent and tried not to ask for help as they did not want to burden their families or health professionals. Some patients hid their suffering, while others used humour to cope. Some felt that they had developed maladaptive coping strategies:

(Patient-3_Follow-up): The way I cope with things is I put them in boxes in my head and shove them away and then switch off, and I need to stop doing that because it is damaging me and it’s damaging my relationship.

Being diagnosed with this life-limiting disease put death in front of patients which triggered them to prepare for it and sort things out before they die. Spiritual practices, such as mindfulness meditation, enabled patients to mitigate the physical impact of heart failure. Some patients searched for information about heart failure to enhance their understanding of their illness and available treatment options. Through this, they were able to discuss treatment options with healthcare professionals and became experts on their condition.

#### Patient support

Multiple health professionals endeavoured to support patients to manage the impact of heart failure. Patients and families did not feel that there were too many health professionals involved in patient care. However, having several health professionals resulted in poor communication and care coordination, conflicts in healthcare decisions, and disintegrated and non-holistic care. Some patients and family carers felt that their General Practitioners (GPs) took a backseat as their care needs were addressed by the specialists:

(Family carer-3_Follow-up): The contact with the (GP) is primarily, it would seem, a kind of function of the bureaucracy of it all, of having to go through a certain person to organise certain other things, rather than it seems to me him adding any value to the process.

Doctors and nurses were mainly involved in addressing the physical symptoms, although nurses also addressed psychological and emotional issues. Hospice teams were highly valued by patients and families as they were key in addressing patient needs and providing holistic care. In contrast, patients and family carers described cases where health professionals outside the hospice failed to address patient care needs because of the lack of time and expertise. Some healthcare professionals did not intervene further to manage advanced heart failure as they were shifting towards a more palliative care approach. Nevertheless, they acknowledged the difficulty in giving up active treatments when medical options are available and found it hard to explain their decision to the patients:

*“(Palliative care clinical nurse specialist): …sometimes it’s harder to do nothing than to do something…*

*(Palliative medicine consultant): […] Cardiologists, because they, you know, they’ve got lots of exciting things Cardiologists can do, so I think that’s… the other thing is, the more things you can possibly do, the more likely you are to do them.” (Group interview-1).*

Caring expectations were placed by patients and health professionals on family carers in particular; some of whom had health issues and needed help themselves. Most families were struggling to cope with patient care and needed support:

*“(Social worker): With long-term illnesses you kind of… you get sucked into the caring role as well, don’t you? So you don’t actually realise the stresses until it can really build up to quite a significant level…” (Group interview-2).*

Poor professional support for families was evident in some cases. This was attributed to the limited resources and poor information sharing about the needs of the family.

### Theme-3: recognising palliative phase

This theme describes the recognition of the palliative phase of patients with heart failure and the subsequent initiation of palliative care conversations with patients and families. A needs-based approach was advocated to identify those requiring palliative care. Once this recognition happened, health professionals engaged with patients and families in palliative care conversations to discuss patient preferences and care plans. However, these conversations were difficult and infrequent because of the prognostic uncertainty of heart failure, patient and family misconceptions of heart failure and palliative care, and lack of time and communication skills of healthcare professionals. The timing of and staff responsible for these conversations were highly debated.

#### Identifying palliative patients

Health professionals advocated a needs-based approach over the prognostic approach for identifying palliative patients because of the unpredictable trajectory of heart failure. They preferred an “in and out” approach to specialist palliative care where it is provided as needed for patients with complex palliative care needs, after which it is withdrawn to allow generalist palliative care services to manage less complex needs. However, patients and family carers preferred to stay in hospices where they got maximal support. They wanted other healthcare professionals to provide a more holistic assessment of their palliative care needs and treat them as unique persons rather than names or numbers:

(Patient-7_Follow-up): I think they’re (consultants) a bit too important for their own importance, and the importance of your actual life and what it’s (heart failure) doing to your life isn’t really an issue for them.

#### Palliative care conversations

Palliative care and end-of-life conversations were considered necessary to discuss patient care plans and help them make decisions in their life:

(Patient-3_Follow-up): It (Advance care planning) was actually quite cathartic because I hadn’t really thought about it, but it let me have my voice. If I couldn’t speak, my voice is down in black and white, so it was actually quite good…

However, some felt these conversations were difficult to conduct. Because of the unpredictable heart failure trajectory, healthcare professionals lacked the confidence to have an end-of-life conversation with deteriorating patients who might get better unexpectedly. Therefore, they waited for the patients to open the conversations, although this rarely happened. The lack of time, communication skills, palliative care knowledge, and experience of healthcare professionals were other perceived barriers to palliative care conversations. At the patient and family level, misperceptions of palliative care (for dying patients) and heart failure (not life-threatening) hindered such conversations.

Patients, families, and health professionals favoured open and honest conversations about diagnosis, prognosis, and available treatment options. However, healthcare professionals were either unable to provide accurate prognostic information or were reluctant to disclose such information to avoid distressing patients:

(Patient-4_Follow-up): Yeah, I would [prefer straight-talking from professionals], yeah, because half the time I come out and I don’t believe what they tell me. […] I think by doing that (telling me the whole story) they are thinking of me really, thinking: Well, if we tell her that, she’ll worry; if we don’t tell her, she won’t know, sort of thing. But I think that’s the wrong thing to do when you’re on your own.

In a few cases, patients and families did not want to know about their diagnosis and prognosis nor talk about advance care planning. Therefore, health professionals called for an individualised and patient-led approach to the conversations; emphasising that not every patient needs all information or “vigorous conversations”.

The timing of palliative care conversations, who broaches it, and how it is followed up had a big impact on patients and families. Health professionals preferred early conversations but they had concerns as patients may improve afterwards and the same conversation has to be repeated:

*“(Physiotherapist): I think, as we touched on before, that with people with COPD and heart failure, because you can’t predict how the disease is going to pan out and what timescale you’ve got, it sometimes is harder to make sure that people are having the right conversations at the right time…” (Group interview-3).*

Palliative care conversations were considered a collective responsibility that should be initiated by one health professional and then communicated to the others to follow them up as patients move between different healthcare settings. Although there was no agreement about who should start the conversations, health professionals felt that GPs or community nurses could be good candidates as “they know the patients best”. However, many conversations were initiated by palliative care specialists, which was perceived as difficult because they are the people dealing with dying.

### Theme-4: coordination of care

This theme describes the organisation of care and communication between health professionals across different care settings to meet the palliative care needs of patients and families along the whole trajectory of heart failure. This communication was perceived to be affected by cultural, organisational, environmental, and financial factors. Patients and families needed continuity of care and consistency of health professionals. Care coordination was deemed necessary, but there was a debate about whose responsibility this is.

#### Networking

Patients and family carers were not completely aware of the connections between the health professionals involved in their care. Poor and one-way communications were perceived to be more common than two-way communications. Patients and family carers indicated a need for integration, communication, and information sharing between all health professionals:

(Family carer-3_Baseline): Sorry, but it’s just a pet hate and I just think it’s… it’s just silly. Like many things, you know, human body and its care and health are a marvellously, complicated thing, but the rest of it, why not just talk to each other… about the right stuff?

Health professionals emphasised the importance of collaborative working, joint palliative care meetings, and joint education to provide good-quality care. The latter includes education about the available care services and their roles to seek advice, refer patients, and avoid conflicts and duplication.

The communication between health professionals was perceived to be affected by several factors. These include cultural differences between health and social care agencies in talking openly about death; difficulties in organising joint home visits; use of different palliative care pathways, policies, and assessment tools; staff consistency; availability of resources; and information-exchange systems. Good communication provided reassurance for patients, developed trust between clinicians, resolved medical decision conflicts, facilitated patient referrals and timely access to healthcare services, and enhanced integrated, patient-centred, and holistic care. Conversely, poor communication made health professionals unaware of what each other is doing; leading to duplication. It created feelings of frustration, worry, isolation, and upset which in turn exacerbated patients’ physical symptoms. Poor information sharing made health professionals unaware of the patient condition, care needs, and current care plan, which triggered patients to act as “conduits of information” and left clinicians acting in contrast to patient preferences.

The communication of health professionals with patients and families was generally poor. Patients wanted to be copied into consultant letters and informed about their test results, appointments, management plan, and changes in their healthcare team. The communication with patients and families was perceived to be affected by environmental factors (better communication in areas with small healthcare teams), availability of resources, how patients perceive the care network around them, and ease of contact:

*“(Lymphoedema nurse specialist): …we’ve discovered patients often have difficulty accessing the GP and, if they’ve got a Specialist Nurse that they’ve built that rapport with and they can rely on, they will ultimately go back to you rather than then going to the GP. You can encourage them to do that but whether they will or not… can be difficult, because they will always go to the person who will give them the answers and things that they want.” (Group interview-2).*

#### Continuity of care

Patients, family carers, and health professionals wanted palliative care to be provided across different healthcare settings from the point of diagnosis, along the whole disease trajectory, into death and bereavement. Patients asked for more frequent monitoring of their condition, long-running responsive services, and caregivers to be available when needed. However, this was not always possible because of the lack of resources. Patients were worried about being “crossed off the lists” of their consultants and heart failure nurse specialists, as their heart failure is “still there” and may relapse anytime. Premature discharge from health and palliative care services resulted in the loss of relationships with health professionals and feelings of abandonment and isolation.

While multiple health professionals were needed to provide continuity of care across different healthcare settings, it was considered important to have consistent health professionals over time. Staff consistency enabled health professionals to work and train together, strengthened their relationships with patients, and increased their awareness of the patient’s condition. Nevertheless, staff turnover was a common problem:

(Family carer-7_Follow-up): And he (GP) died a few weeks ago, but they (surgery) haven’t sent a letter saying ‘You are now under this doctor,’ because they don’t have any… they don’t have any long-time doctors. (GP1), they’re all locums and come-and-go doctors, so you don’t know who the heck you’re seeing. You haven’t a clue.

Patients, family carers, and health professionals asked for a care coordinator who would organise patient care and act as a single point of contact for patients to communicate their healthcare needs to other health professionals, ensure that their care preferences are met, and ensure that timely palliative care is delivered at the right level. However, this role was lacking and there was no common understanding of whose responsibility this is. Some health professionals thought it would be unrealistic to allocate one identified key worker who follows the patient throughout the whole course of their illness. Others suggested that GPs, district nurses, or palliative care clinical nurse specialists could be the care coordinators as they spend more time with patients. Where no care coordinator was available, patients or their families took the responsibility to organise patient care:

(Patient-5_Joint interview_Baseline): Where do I fit in? Oh, I’m Queen Bee, sitting in the middle. [All chuckle] I mean I set it up. I decided because… I used to be what they called ‘a good secretary’. I set things up and I organise and I’m good at human resources. You might not think so: you might I’m argumentative…

## Discussion

This study aimed to evaluate the perceptions of patients with advanced heart failure, family carers, and health professionals on the holistic palliative care needs of the patients and their families and how, and by whom, they can be addressed. Four themes were generated to address the research objectives: Impact of heart failure, Coping and support, Recognising palliative phase, and Coordination of care. Other qualitative studies that assessed patients’ lived experiences of heart failure and palliative care provision described similar themes [28, 30, 46, 47], although details on how they relate to each other were mostly lacking. Low et al. suggested that poor multidisciplinary communication and care coordination (equivalent to Theme-4) and lack of confidence to engage in open palliative care conversations (equivalent to Theme-3) may affect the quality of the provided care (equivalent to Theme-2) and thus fail to relieve the multidimensional impact of heart failure (equivalent to Theme-1) [46]. Likewise, in this study, the four themes were illustrated in a thematic map that shows how heart failure impact (Theme-1) could be relieved through three co-existing interrelated factors (Themes-2,3,4). Although the thematic map suggests that these factors have the same impact, it was not possible to determine, from the data available, the degree of impact of each factor.

The palliative care needs of patients and family carers are commonly identified in other studies [28–30, 46, 47], though spiritual needs were less evident. To address the impact of heart failure on patients and family carers, a holistic assessment of patient and family palliative care needs is needed [48]. This could be facilitated by using a comprehensive needs-assessment tool such as the Needs Assessment Tool: Progressive Disease - Heart Failure (NAT: PD-HF) [49, 50]. Such a tool can be used by healthcare professionals during routine patient assessment in the clinic to identify, assess the severity, and trigger actions for their palliative care needs.

Nurses played a role in managing patient and family palliative care needs, but it was the hospices that provided more holistic support, while GPs had a little contribution. In other studies, palliative care generalists were expected to have the appropriate skills to address patient needs, but this was hampered by the lack of training in symptom control and palliative care [31, 46], which is also observed in this study. Patients attributed some cases of medical failure to forgoing curative options by healthcare professionals and shifting towards palliative care, which reflected a lack of patient understanding of heart failure and palliative care. To address these issues, palliative care generalists should be trained on palliative care needs assessment and management, while patients should be educated about heart failure and palliative care.

Introducing palliative care to patients with heart failure is complicated by the lack of palliative care skills and knowledge, confusing palliative care with end-of-life care, misperceptions of palliative care referral as a medical failure, and patients’ unawareness of their prognosis [31]. The biggest hurdle that emerged from the interviews was how, when, and by whom the palliative care conversations should be conducted. While open and early conversations are advocated to help patients deal with personal affairs, some healthcare professionals are still reluctant to engage in such discussions for fear of reducing hope and triggering anxiety [46]. The interviewed health professionals expected GPs and community nurses to initiate the conversations, but both heart failure nurse specialists and cardiologists can also be good candidates based on their knowledge of heart failure and relationship with patients [46]. Regardless of who is conducting the conversations, they should be trained to enhance their palliative care communication skills.

The poor communication and lack of continuity of care shown in this study are commonly cited in the heart failure literature [29, 30, 47]. Using a needs-assessment tool like NAT:PD-HF could facilitate communication as healthcare professionals complete the tool in the clinics to discuss patients’ palliative care needs and share it with other healthcare professionals [50, 51]. Poor communication resulted in fragmented, uncoordinated care. The role of a care coordinator was lacking and there were different perspectives on whose responsibility this is. In other studies, while patients were willing to have a single care coordinator, health professionals thought that this might be the role of nurses or doctors or even a collective responsibility [31, 46]. Research is needed to further explore this issue.

### Strengths and limitations

Although previous heart failure studies reported experiences with palliative care [31, 46], most studies focused on the perspectives of healthcare professionals, rather than those of patients receiving palliative care and their family carers. This study explored the perspectives of patients, family carers, and health professionals from multiple services, which added richness, depth, and detail to the data. Besides, the interviews were conducted longitudinally, which enabled comparing patients’ palliative care needs and professional support over time. All patients and family carers interviewed at baseline completed their follow-up interview, apart from patients who had died after their baseline interview.

Merging patient, family carer, and health professional interviews to generate the themes added breadth to the analysis. The combination of interview data enabled exploring and comparing multiple perspectives on palliative care services, provided a comprehensive view of patients’ palliative care needs, and produced a more complete picture and better understanding of palliative care experiences. However, as the interview topic guides for patient and family carer interviews were slightly different from those for health professional interviews [32], the first two themes were more prominent in the patient and family carer interviews, while the last two were more prominent in the health professional interviews.

Exploring different perspectives of participants from multiple centres enhances the transferability of the study findings [31]. However, only the data collected inside the UK were analysed for this study because of the lack of access to data in other countries and differences in healthcare structures [52]. Consequently, the findings may not be transferable to countries with different healthcare structures.

A methodological strength of the study is the use of Normalisation Process Theory as a theoretical framework for the data analysis to aid in understanding the experiences with implementing palliative care. Normalisation Process Theory is commonly used in the health literature to understand implementation processes and individuals’ experiences [36, 44], but its use in the palliative care field is scarce [53–55]. One concern is that some data may fall outside the predetermined Normalisation Process Theory constructs [36, 56]. This issue was mitigated by adopting a mainly inductive approach in the secondary data analysis, though seen through the lens of Normalisation Process Theory.

Secondary qualitative data analysis has some challenges. One issue is that the data collected for the primary study may not fit the purpose of the secondary study [57, 58]. By examining the primary study, it was found congruent with this study’s focus and objectives. A second challenge concerns the quality and completeness of the primary data [59]. This was checked using the Assessment Tool: Criteria for Use in a Secondary Analysis of Qualitative Data [59] (Additional file 3). Briefly, the audio-recorded interviews were conducted by well-trained, experienced researchers and had a clear agenda, while the interview transcripts were comprehensible and interpretable.

A third challenge of secondary qualitative data analysis concerns the currency of the primary dataset, as some contexts may change with time [59]. Although the interviews were conducted five years before the secondary data analysis, the practice of palliative care for patients with heart failure in the UK study sites has not changed considerably since then [60]. A final issue is that the secondary analyst interprets data which were collected by other researchers and co-constructed through their interaction with participants [57, 61]. Although BR was not involved in the primary study, one of the study team (NP) was part of the original study. NP familiarised BR with the study context and signposted him to relevant journal papers from the primary study. Ultimately, the secondary analysis aimed to recontextualise and reconstruct data and provide new insights, rather than recreating the primary study context [62].

## Conclusions

The study provided broad insight into the experiences of patients with advanced heart failure, family carers, and health professionals with palliative care services. It showed the impact of heart failure on patients and their families, how they cope, and how they could be supported to address their palliative care needs. It highlighted the difficulty in recognising the palliative phase and conducting palliative care conversations and the importance of coordinating care among healthcare teams. The study findings can help researchers and healthcare professionals to design palliative care interventions focusing on the perceived care needs of patients and families. Such interventions should move beyond optimising the medical treatment for patients with heart failure to a more holistic, person-centred approach that would improve the quality of life of patients and family carers. The use of a palliative care needs-assessment tool is recommended to enable holistic assessments and facilitate palliative care conversations. Future palliative care interventions should focus on enhancing the communication skills of healthcare professionals, training them on palliative care, and educating patients about advanced heart failure and what palliative care could offer.

### Electronic supplementary material

Below is the link to the electronic supplementary material.

Supplementary Material 1

Supplementary Material 2

Supplementary Material 3

## Data Availability

The datasets analysed during the current study are available from the corresponding author on reasonable request.

## Abbreviations

COPD: Chronic Obstructive Pulmonary Disease
GP: General Practitioner
InSup-C: Integrated Palliative Care in Cancer and Other Chronic Conditions
NAT: PD-HF: Needs Assessment Tool:Progressive Disease - Heart Failure
