# Supplementary material for: The experiences of patients with advanced heart failure, family carers, and health professionals with palliative care services: a secondary reflexive thematic analysis of longitudinal interview data
Source: BMC Palliat Care. 2023 Aug 10;22:115. doi: 10.1186/s12904-023-01241-1 (PMC10413510; doi:10.1186/s12904-023-01241-1)
Supplement: Supplementary file 1 — Supplementary Material 1 [file 12904_2023_1241_MOESM1_ESM.pdf]

Interview protocol: Baseline interview patient

What to bring?
- big white paper<br>- pens for making notes and for marking the collaboration between caregiver in theme 2.<br>- post-its<br>- audio-recorder + extra batteries<br>- photo camera + extra batteries<br>- note book<br>- participant information sheet (PIS)<br>- demographic questions<br>- social network analysis (SNA) questionnaire

Introduction

1. Who is the interviewer?

- Who you are and where you are from.

2. What can I and/or can I not do for you?

- As a researcher you are here to ask questions in light of the study.
- You are not (here) a(s) caregiver and therefore cannot give advice about aspects related to the patient's illness. (If necessary you can instruct/support the patient to contact his/her caregiver).
- You have about 1.5 hours. Does the patient have any appointments after this interview which you should take into account?
- Whenever the patient is not feeling well, (s)he can decide to interrupt or stop the interview at any moment.
- Explain what the patient can expect during this contact moment: (1) general introduction about aim and procedures of entire study; 2) first interview (comprising demographic data, SNA and the interview itself).

3. Participant information sheet (reiteration)

The following items of the PIS should be reiterated:

- Aims of the study (see PIS)
- Study procedures/follow-up (see PIS)
- Reason why you want to interview the patient (see PIS)
- Reason why personal information of the patient is necessary (importance of seeing the results in the right context).
- You are interested in the personal view of the patient and that there are no (right or) wrong answers.
- Ask permission to audio-record the interview (see PIS) and make pictures. Explain why this is necessary.
- The information the patient provides is confidential (see PIS).
  (You could mention that all quotes / details that may be used in presentations or papers do

An EU Framework 7 Programme (FP7/2007-2013) under grant agreement n° 335555

not use names and initials etc. of patients (= kept confidential).
- Explain why will you make notes during the interview.

4. What are the aims and procedure of this baseline interview?

a) To explore how the patient experiences the care and support (s)he receives from caregivers for his/her problems and needs.
b) To know more about how (s)he experiences contact with caregivers and collaboration between caregivers.

- Before the actual interview starts, you will ask some personal, demographic questions. Secondly you will conduct a social network analysis (in order to get a picture of the caregivers that are around the patient to give care or support).
  Then you will start the interview in which you will explore the answers given in the social network analysis. You will use cards to structure the interview questions. In the interview you will ask various questions about these cards. Invite the patient to think out loud why (s)he is placing the cards in a particular way.

5. Assess demographic data

[Be aware of problems / needs the patient may raise here and during SNA and make notes of these. Come back to these during the discussion of problems/needs in the interview if the patient does not mention these then].

6. Conduct the Social Network Analysis (SNA)

[Write down the caregivers the patient has contact with on cards (use SNA). Also make a card with 'patient' on it. Place these cards in front of the patient]. Before you are going to use these caregiver cards, you will ask some questions about problems and needs. Explain that the interview will start now.

➔ **Start audiotape**

<u>The interview</u>

Theme 1: problems and needs

Introduce the theme 'problems and needs'. Experiences of patients with provision of care and contacts with caregivers, often have to do with what they expect of it, whether it meets their expectations and their problems and needs. Therefore you would like to explore illness related problems/needs the patient may experience. When you have a picture of the patients' problems and needs, you will explore how the patient experiences his/her contact with caregivers and the collaboration between caregivers. [Explain the difference between problems and needs].

Problems

Introduce the questions about problems: e.g. "given that your are ill, I could imagine that you

An EU Framework 7 Programme (FP7/2007-2013) under grant agreement n° 335555

experience various problems...”

Questions	Probing opportunities
**Q1: Can you tell which problems you currently encounter?**<br><br>[Write down each problem on a card]	1. *In case the patient does not know how to express problems, you can ask for problems in the following domains:*<br>- *Daily activities*<br>- *Problems with your body*<br>- *Problems with(in) your mind*<br>- *Administrative (and financial) matters*<br>- *Family/friends matters*<br>- *Receiving support (what or who?)*<br>- *Independency*<br>- *Need for information*<br>- *The care you receive*<br><br>2. *When patients only mentions a vague term, e.g. ‘fear’:*<br>- *What are you scared for?*<br>- *Can you tell about a situation in which you were scared?*<br>- *What do you do when you are scared?*<br>- *What does fear do with you?*<br><br>3. *In case the patient does not experience any problems, you can ask: “Which problems (related to the illness) you experienced in the past are currently supported?”*
**Q2: Can you place these problem cards in order of importance to you?**	
**Q3: Can you place these problems cards in order of priority (which problems need to be resolved by a caregiver first of all)?**<br><br>[Take a picture of the problems in order of priority]	- *Explore why the patient chooses this particular order.*
**Summarise**	

Needs

After discussing the problems, you will now ask the same questions for needs.

Questions	Probing opportunities
**Q4: Can you tell which needs you experience: i.e. issues for which you need support/attention? (write down each need on a card).**	*When e.g. 'being in control' is mentioned:*<br>- *What happens when you are in control?*<br>- *Can you tell about a situation in which you were in control?*<br>- *What does it mean for you to be in control?*
**Q5: Can you place these cards in order of priority (which of these needs need to be resolved by a caregiver first of all)?**<br><br>[Take a picture of the needs in order of priority]	- *Explore why the patient chooses this particular order.*
**Summarise**	

Theme 2: relationships and communication with and between caregivers

Relations between the patient and caregivers

Explain that you would like to explore the contact between the patient and caregivers now. You are going to use the cards with caregivers on it. Place the 'patient' card in the middle.

Questions	Probing opportunities
**Step 1: With which caregivers do you have more or less frequent contact? (refer to the SNA)**<br><br>[Let the patient place the caregivers with whom (s)he has most frequent contact closest to the 'patient' card and those with whom (s)he has the least frequent contact further away from the 'patient' card]<br><br>[Take a picture of the frequency of contacts in the care network]	
**Step 2: *What* do these caregivers do when you have contact with them?**	- *What kind of care or support is provided?*

**Step 3: *Which* caregivers are most important to you?**<br><br>[Let the patient place the caregivers which are more important for him/her closer to the patient card and those who are less important further away].<br><br>[Take a picture showing the caregivers that are more and less important for the patient]	*You can think of importance in two dimensions:*<br><br>- *Relational dimension*<br>*e.g. caregiver shows acknowledgement (has emphatic attitude); shows commitment; shows involvement*<br>- *Giving dimension*<br>*e.g. caregiver gives medication/ treatment; gives information (by being clear/honest about the patient's current position and/or prognosis); gives hope*
**Step 4: *Why* are these caregivers important to you?**	
**Step 5: For which of the 3 highest priority problems and needs (Q3 and Q5) support is currently provided by these caregivers?**<br><br>[Place the card with the problems/needs on the particular caregiver. If there are more caregivers that treat one problem, make more cards with the same problem on it].	
**Step 6: Would you like to mention an additional priority problem/need for which you currently receive support, that you did not mention in step 5?**<br><br>[Take picture showing the 3 (or 4) highest priority problems/needs that are currently supported by caregivers and the problems/needs that remain]	- *Explore why this additional problem/need is mentioned.*
**Summarise**	

Collaboration between caregivers

Explain that after discussing the patient's contact with several caregivers, you would like to focus on the collaboration between caregivers. Let the patient draw lines between caregivers using markers.

Questions	Probing opportunities
**Q11: Who, do you think, works together in the care network in front of you. How do you notice this?**<br><br>[Take a picture showing collaboration between caregivers in the network]	- *Explore whether the collaboration is focused on care for the patient or on care for the family caregiver.*
**Q12: What is the importance for you, that these caregivers work together? Why?**	
**Q13: Who, do you think, should work together but does not do this now? Why?**<br><br>[Take a picture showing caregivers in the network that should work together]	- *Explore why they do not work together now.*
**Summarise**	
**Is there something you would like to say or add?**<br><br>**Do you have any questions?**	

Closing

Thank the patient for his/her time and if applicable, give present.

Explain what will happen from now on:

- Keep diary weekly
- Complete questionnaires monthly
- That you will remind the patient (by telephone?) to fill in the diary and questionnaires.
- The results will be available at the end of 2015/early 2016. In the meantime patient can visit website www.insup-c.eu.
- Give your contact details in case the patient wishes to contact you or has questions.

➔ **Stop audiotape**

Interview protocol: Final interview patient

What to bring
- photos of baseline interview!<br>- big white paper (1 from baseline interview + 1 empty paper)<br>- post-its (from baseline interview + empty ones)<br>- pens for making notes and for marking the collaboration between caregiver in theme 2.<br>- audio-recorder + extra batteries<br>- photo camera + extra batteries<br>- note book<br>- demographic question(s)<br>- social network analysis (SNA) questionnaire<br>- patient diary<br>- extra questions about burden of interviewing

Introduction

1. Building rapport after the first interview + practical matters

- Ask the patient how (s)he is (building rapport).
- Mention that whenever the patient is not feeling well (s)he can decide to stop the interview at any moment.
- Mention that that are no right or wrong answers.

2. What is the aim of this final interview?

- You have 'followed' the patient with a social network analysis, questionnaires, diary and interview.
- You will look back on the previous 3 months and you are interested in what is still the same and what has changed regarding problems/needs, contact with and collaboration between caregivers.
- You will make use of the photos and the white paper you made in the baseline interview and you will use the 'card game' again. Invite the patient to think out loud again when (s)he is placing the cards in his/her care network.
- You will first ask question 8 of the demographics (performance status patient) and conduct the social network analysis. Subsequently you will start with the actual interview. After the interview you will ask the patient to answer a few written questions about the possible burden/reward of being interviewed (except for UK)

3. Ask question 8 of the demographics (performance status patient).

[Be aware of problems / needs the patient may raise here and during SNA and make notes of these. Come back to these during the discussion of problems/needs in the interview if the patient does not mention these then].

An EU Framework 7 Programme (FP7/2007-2013) under grant agreement n° 335555

4. **Conduct the Social Network Analysis (SNA)**

[Write down the caregivers the patient has contact with on cards (use SNA). Also make a card with 'patient' on it. Place these cards in front of the patient].

➔ **Start audiotape**

<u>The interview</u>

Theme 1: problems and needs

In the baseline interview you discussed problems and needs experienced by the patient. Explain that you could imagine that some of the problems / needs are still the same and some may have changed (worsened/diminished) in comparison to 3 months ago. You will start this theme by following the same procedure as in the baseline interview to explore current problems and needs. Then you will show the picture of the baseline interview showing problems and needs, and you will ask for the differences in current problems and needs compared to those of 3 months ago. [If necessary explain the difference between problems and needs again].

An EU Framework 7 Programme (FP7/2007-2013) under grant agreement n° 335555

Problems

Questions	Probing opportunities
**Q1: Can you tell which problems you currently encounter?**<br><br>[Write down each problem on a card]	1. *In case the patient does not know how to express problems, you can ask for problems in the following domains:*<br>- *Daily activities*<br>- *Problems with your body*<br>- *Problems with(in) your mind*<br>- *Administrative (and financial) matters*<br>- *Family/friends matters*<br>- *Receiving support (what or who?)*<br>- *Independency*<br>- *Need for information*<br>- *The care you receive*<br><br>2. *When patients only mentions a vague term, e.g. 'fear':*<br>- *What are you scared for?*<br>- *Can you tell about a situation in which you were scared?*<br>- *What do you do when you are scared?*<br>- *What does fear do with you?*<br><br>3. *In case the patient does not experience any problems, you can ask: "Which problems (related to the illness) you experienced in the past are currently supported?"*
**Q2: Can you place these problem cards in order of importance to you?**	
**Q3: Can you place these problems cards in order of priority (which problems need to be resolved by a caregiver first of all)?**<br><br>[Take a picture of the problems in order of priority]	- *Explore why the patient chooses this order.*
**Summarise**	

Needs

After discussing the problems, you will now ask the same questions for needs.

Questions	Probing opportunities
**Q4: Can you tell which needs you experience: i.e. issues for which you need support/attention? (write down each need on a card).**	*When e.g. 'being in control' is mentioned:*<br><br>- *What happens when you are in control?*<br>- *Can you tell about a situation in which you were in control?*<br>- *What does it mean for you to be in control?*
**Q5: Can you place these cards in order of priority (which of these needs need to be resolved by a caregiver first of all)?**<br><br>[Take a picture of the needs in order of priority]	- *Explore why the patient chooses this order.*
**Summarise**	

Comparison of problems and needs to 3 months ago

Take the picture with problems/needs discussed in the baseline interview and show it to the patient.

Questions	Probing opportunities
**Q6: If you compare the problems and needs you currently encounter to those on the picture, which problems and needs have changed and which remained the same?**	- *Explore the differences and similarities.*
**Summarise**	

Theme 2: relationships and communication with and between caregivers

Relations between the patient and caregivers

Explain that you would like to explore the current contact between the patient and caregivers now. Again you will follow the same steps as in the baseline interview. Then you will compare the current situation to that of 3 months ago.

You are going to use the cards with caregivers on it. Place the 'patient' card in the middle.

An EU Framework 7 Programme (FP7/2007-2013) under grant agreement n° 335555

Questions	Probing opportunities
**Step 1: With which caregivers do you have more orless frequent contact? (refer to the SNA)**<br><br>[Let the patient place the caregivers with whom (s)he has most frequent contact closest to the ‘patient’ card and those with whom (s)he has the least frequent contact further away from the ‘patient’ card]<br><br>[Take a picture of the frequency of contacts in the care network]	
**Step 2: *What* do these caregivers do when you have contact with them?**	- *What kind of care or support is provided?*
**Step 3: *Which* caregivers are most important to you?**<br><br>[Let the patient place the caregivers which are more important for him/her closer to the patient card and those who are less important further away].<br><br>[Take a picture showing the caregivers that are more and less important for the patient]	*You can think of importance in two dimensions:*<br><br>- *Relational dimension e.g. caregiver shows acknowledgement (has emphatic attitude); shows commitment; shows involvement*<br>- *Giving dimension e.g. caregiver gives medication/ treatment; gives information (by being clear/honest about the patient’s current position and/or prognosis); gives hope*
**Step 4: *Why* are these caregivers important to you?**	

An EU Framework 7 Programme (FP7/2007-2013) under grant agreement n° 335555

**Step 5: For which of the 3 highest priority problems and needs (Q3 and Q5) support is currently provided by these caregivers?**<br><br>[Place the card with the problems/needs on the particular caregiver. If there are more caregivers that treat one problem, make more cards with the same problem on it].	
**Step 6: Would you like to mention one additional priority problem/need for which you currently receive support, that you did not mention in step 5?**<br><br>[Take picture showing the 3 (or 4) highest priority problems/needs that are currently supported by caregivers and the problems/needs that remain]	- *Explore why this additional problem/need is mentioned.*
**Summarise**	

Comparison of relations between patient and caregivers to the situation 3 months ago

Take the picture with the patient-caregiver network discussed in the baseline interview and show it to the patient.

Questions	Probing opportunities
**Step 7: If you compare the caregivers with whom you currently have contact and what they do to those on the picture, what has changed and what remained the same?**	- *Explore the differences and similarities.*
**Summarise**	

Collaboration between caregivers

Explain that after discussing the patient's contact with several caregivers, you would like to focus on the collaboration between caregivers. Let the patient draw lines between caregivers using markers. You will follow the same procedure as in the baseline interview and will subsequently ask for the differences and similarities compared to 3 months ago.

An EU Framework 7 Programme (FP7/2007-2013) under grant agreement n° 335555

Questions	Probing opportunities
**Q11: Who, do you think, works together in the care network in front of you. How do you notice this?**<br><br>[Take a picture showing collaboration between caregivers in the network]	- *Explore whether the collaboration is focused on care for the patient or on care for the family caregiver.*
**Q12: What is the importance for you, that these caregivers work together? Why?**	
**Q13: Who, do you think, should work together? Why?**<br><br>[Take a picture showing caregivers in the network that should work together]	- *Explore why do they not work together now.*
**Summarise**	
**- Is there something you would like to say or add?**<br><br>**- Do you have any questions?**	

Comparison of collaboration between caregivers to 3 months ago

Take the picture about collaboration between caregivers in the network discussed in the baseline interview and show it to the patient.

Questions	Probing opportunities
**Q6: If you compare the caregivers who do and do not work together to those on the picture, what has changed and what remained the same?**	- *Explore the differences and similarities.*
**Summarise**	
**Is there something you would like to say or add?**<br><br>**Do you have any questions?**	

An EU Framework 7 Programme (FP7/2007-2013) under grant agreement n° 335555

Closing

[Collect the names (+ contact details if possible) of the professional caregivers involved in the patient's care network (these caregivers will be invited for the focus group)].

Thank the patient for his/her time.

Explain what will happen from now on and how the patient can come to know more about the results (The results will be available at the end of 2015/early 2016. In the meantime patient can visit website www.insup-c.eu).

➔ **Stop audiotape**

An EU Framework 7 Programme (FP7/2007-2013) under grant agreement n° 335555

Interview protocol: Baseline interview family caregiver

What to bring?
- big white paper<br>- pens for making notes and for marking the collaboration between caregiver in theme 2.<br>- post-its<br>- audio-recorder + extra batteries<br>- photo camera + extra batteries<br>- note book<br>- participant information sheet (PIS)<br>- demographic questions

Introduction

1. Who is the interviewer?

- Who you are and where you are from.

2. What can I and/or can I not do for you?

- As a researcher you are here to ask questions in light of the study.
- You are not (here) a(s) caregiver and therefore cannot give advice about aspects related to the patient's illness. (If necessary you can instruct/support the family caregiver / patient to contact the caregiver).
- You have about 1.5 hours. Does the family caregiver have any appointments after this interview which you should take into account?
- Whenever the family caregiver is not feeling well, (s)he can decide to interrupt or stop the interview at any moment.
- Explain what the family caregiver can expect during this contact moment (1) General introduction about aim and procedures of entire study. 2) Interview starting with demographic data, and then the interview itself).

3. Participant information sheet (reiteration)

The following items of the PIS should be reiterated:

- Aims of the study (see PIS)
- Study procedures/follow-up (see PIS)
- Reason why you want to interview the family caregiver (see PIS)
- Reason why personal information of the family caregiver is necessary (importance of seeing the results in the right context).
- You are interested in the personal view of the family caregiver and that there are no (right or) wrong answers.
- Ask permission to audio-record the interview (see PIS) and make pictures. Explain why this is necessary.
- The information the family caregiver provides is confidential (see PIS).
  (You could mention that all quotes / details that may be used in presentations or papers do not use names and initials etc. of patients/family caregivers (= kept confidential).

An EU Framework 7 Programme (FP7/2007-2013) under grant agreement n° 335555

- Explain why will you make notes during the interview.

4. **What are the aims and procedure of this baseline interview?**

a) To explore how the family caregiver experiences the care and support provided by professional caregivers for the problems and needs of the patient.
b) To know more about how the family caregiver experiences the contact between the patient, him/herself, and professional caregivers and collaboration between caregivers.

- Before the actual interview starts, you will ask some personal, demographic questions. Then you will start the interview. You will use cards to structure the interview questions. In the interview you will ask various questions about these cards. Invite the family caregiver to think out loud and explain why (s)he is placing the cards in a particular way.

5. **Assess demographic data**

[Be aware of problems / needs the family caregiver may raise here and during SNA and make notes of these. Come back to these during the discussion of problems/needs in the interview if the family caregiver does not mention these then].

➔ **Start audiotape**

<u>The interview</u>

Theme 1: problems and needs

Introduce the theme 'problems and needs'. Experiences of family caregivers/patients with provision of care and contacts with professional caregivers, often have to do with what they expect of it, whether it meets their expectations and their problems and needs. Therefore you would like to explore illness related problems/needs the patient may experience. When you have a picture of the problems and needs, you will explore how the family caregiver experiences his/her contact with caregivers and collaboration between caregivers. [Explain the difference between problems and needs].

Problems

Introduce the questions about problems: e.g. "given that the patient is ill, I could imagine that (s)he experiences various problems..."

An EU Framework 7 Programme (FP7/2007-2013) under grant agreement n° 335555

Questions	Probing opportunities
**Q1: Can you tell which problems the patient currently encounters?**<br><br>[Write down each problem on a card]	1. *In case the family caregiver does not know how to express problems, you can ask for problems in the following domains:*<br>- *Daily activities*<br>- *Problems with the patient's body*<br>- *Problems with(in) the patient's mind*<br>- *Administrative (and financial) matters*<br>- *Family/friends matters*<br>- *Receiving support (what or who?)*<br>- *Independency*<br>- *Need for information*<br>- *The care the patient receives*<br><br>2. *When family caregiver only mentions a vague term, e.g. 'fear':*<br>- *What is the patient scared for?*<br>- *Can you tell about a situation in which the patient was scared?*<br>- *What does the patient do when (s)he is scared?*<br>- *What does fear do with the patient?*<br><br>3. *In case the patient does not experience any problems according to the family caregiver, you can ask:*<br>*"Which problems (related to the illness) the patient experienced in the past are currently supported?"*
**Q2: Can you place these problem cards in order of importance to him/her?**	
**Q3: Can you place these problems cards in order of priority (which problems need to be resolved by a caregiver first of all)?**<br><br>[Take a picture of the problems in order of priority]	- *Explore why family caregiver chooses this order.*
**Summarise**	

Needs

After discussing the problems, you will now ask the same questions for needs.

An EU Framework 7 Programme (FP7/2007-2013) under grant agreement n° 335555

Questions	Probing opportunities
**Q4: Can you tell which needs the patient experiences: i.e. issues for which (s)he needs support/attention? (write down each need on a card).**	*When e.g. 'being in control' is mentioned:*<br>- *What happens when the patient is in control?*<br>- *Can you tell about a situation in which the patient was in control?*<br>- *What does it mean for the patient to be in control?*
**Q5: Can you place these cards in order of priority (which of these needs need to be resolved by a caregiver first of all)?**<br><br>[Take a picture of the needs in order of priority]	- *Explore why the family caregiver chooses this order.*
**Summarise**	

Theme 2: relationships and communication with and between caregivers

Relations between the patient and caregivers

Explain that you would like to explore the contact between the patient and caregivers and between the family caregiver and professional caregivers now. You are going to use the cards with caregivers on it. First place the 'patient' card in the middle.

An EU Framework 7 Programme (FP7/2007-2013) under grant agreement n° 335555

Questions	Probing opportunities
**Step 1: With which caregivers does the patient have more or less frequent contact? (refer to the SNA)**<br><br>[Let the family caregiver place the caregivers with whom the patient has most frequent contact closest to the 'patient' card and those with whom (s)he has the least frequent contact further away from the 'patient' card]<br><br>[Take a picture of the frequency of contacts in the care network]	
**Step 2: *What* do these caregivers do when the patient has contact with them?**	- *What kind of care or support is provided?*
**Step 3: *Which* caregivers are most important to him/her?**<br><br>[Let the family caregiver place the caregivers which are more important for the patient closer to the patient card and those who are less important further away].<br><br>[Take a picture showing the caregivers that are more and those that are less important for the patient]	*You can think of importance in two dimensions:*<br><br>- *Relational dimension*<br>*e.g. caregiver shows acknowledgement (has emphatic attitude); shows commitment; shows involvement*<br>- *Giving dimension*<br>*e.g. caregiver gives medication/ treatment; gives information (by being clear/honest about the patient's current position and/or prognosis); gives hope*
**Step 4: *Why* are these caregivers important to him/her?**	

An EU Framework 7 Programme (FP7/2007-2013) under grant agreement n° 335555

**Step 5: For which of the 3 highest priority problems and needs (Q3 and Q5) support is currently provided by these caregivers?**<br><br>[Place the card with the problems/needs on the particular caregiver. If there are more caregivers that treat one problem, make more cards with the same problem on it].	
**Step 6: Would you like to mention one additional priority problem/need for which the patient currently receives support, that you did not mention in step 5?**<br><br>[Take picture showing the 3 (or 4) highest priority problems/needs that are currently supported by caregivers and the problems/needs that remain]	- *Explore why this additional problem/need is mentioned.*
**Summarise**	

Relations between the family caregiver and professional caregivers

Now place the card with family caregiver on the patient card.

Questions	Probing opportunities
**Repeat steps 1, 2, 3, 4 in order to examine how the family caregiver experiences his/her own contact with caregivers in the patient's network and to discover if there are differences in contact the patient and family caregiver have with caregivers.**<br><br>[Take a picture of the contacts of the contacts between the family caregiver and professional caregivers].	- *Explore the differences between the family caregiver's and patient's contact (frequency / importance) with professional caregivers.*

Collaboration between caregivers

Explain that after discussing the patient's contact with several caregivers, you would like to focus on the collaboration

An EU Framework 7 Programme (FP7/2007-2013) under grant agreement n° 335555

between caregivers. Let the patient draw lines between caregivers using markers.

Questions	Probing opportunities
**Q11: Who, do you think, works together in the care network in front of you. How do you notice this?**<br><br>[Take a picture showing collaboration between caregivers in the network]	- *Explore whether the collaboration is focused on care for the patient or on care for the family caregiver.*
**Q12: What is the importance for the patient, that these caregivers work together? Why?**	
**Q13: Who, do you think, should work together? Why?**<br><br>[Take a picture showing caregivers in the network that should work together]	- *Explore why they do not work together now according to the family caregiver.*
**Summarise**	
**Is there something you would like to say or add?**<br><br>**Do you have any questions?**	

Closing

Thank the family caregiver for his/her time and if applicable, give present.
Explain what will happen from now on:

- Complete questionnaires monthly
- That you will remind the family caregiver (by telephone?) to fill in the questionnaires.
- The results will be available at the end of 2015/early 2016. In the meantime family caregiver can visit website www.insup-c.eu.
- Give your contact details in case the family caregiver wishes to contact you or has questions.

➔ **Stop audiotape**

An EU Framework 7 Programme (FP7/2007-2013) under grant agreement n° 335555

Interview protocol: Final interview family caregiver

What to bring
- photos of baseline interview!<br>- big white paper (1 from baseline interview + 1 empty paper)<br>- post-its (from baseline interview + empty ones)<br>- pens for making notes and for marking the collaboration between caregiver in theme 2.<br>- audio-recorder + extra batteries<br>- photo camera + extra batteries<br>- note book<br>- pen/pencil<br>- demographic question(s)

Introduction

1. **Building rapport after the first interview + practical matters**

- Ask the family caregiver how (s)he is (building rapport).
- Mention that whenever the family caregiver is not feeling well (s)he can decide to stop the interview at any moment.
- Mention that that are no (right or) wrong answers.

2. **What is the aim of this final interview?**

- You have 'followed' the family caregiver with questionnaires and an interview.
- You will look back on the previous 3 months and you are interested in what is still the same and what has changed regarding problems/needs, contact with and collaboration between caregivers.
- You will make use of the photos and the white paper you made in the baseline interview and you will use the 'card game' again. Invite the family caregiver to think out loud again when (s)he is placing the cards in the care network.

➔ **Start audiotape**

The interview

Theme 1: problems and needs

In the baseline interview you discussed problems and needs experienced by the patient from the family caregiver's standpoint. Explain that you could imagine that some of the problems / needs are still the same and some may have changed (worsened/diminished) in comparison to 3 months ago. You start this theme following the same procedure as in the baseline interview to explore current problems and needs. Then you will show the picture of the baseline interview showing problems and needs, and you will ask for the differences in current problems and needs compared to those of 3 months ago. [If necessary explain the difference between problems and needs again].

An EU Framework 7 Programme (FP7/2007-2013) under grant agreement n° 335555

Problems

Questions	Probing opportunities
**Q1: Can you tell which problems the patient currently encounters?**<br><br>[Write down each problem on a card]	1. *In case the family caregiver does not know how to express problems, you can ask for problems in the following domains:*<br>- *Daily activities*<br>- *Problems with the patient's body*<br>- *Problems with(in) the patient's mind*<br>- *Administrative (and financial) matters*<br>- *Family/friends matters*<br>- *Receiving support (what or who?)*<br>- *Independency*<br>- *Need for information*<br>- *The care the patient receives*<br><br>2. *When family caregiver only mentions a vague term, e.g. 'fear':*<br>- *What is the patient scared for?*<br>- *Can you tell about a situation in which the patient was scared?*<br>- *What does patient do when (s)he is scared?*<br>- *What does fear do with the patient?*<br><br>3. *In case the patient does not experience any problems according to the family caregiver, you can ask:*<br>*"Which problems (related to the illness) the patient experienced in the past are currently supported?"*
**Q2: Can you place these problem cards in order of importance to the patient?**	
**Q3: Can you place these problems cards in order of priority (which problems need to be resolved by a caregiver first of all)?**<br><br>[Take a picture of the problems in order of priority]	- *Explore why the family caregiver chooses this the order.*
**Summarise**	

An EU Framework 7 Programme (FP7/2007-2013) under grant agreement n° 335555

Needs

After discussing the problems, you will now ask the same questions for needs.

Questions	Probing opportunities
**Q4: Can you tell which needs the patient experiences: i.e. issues for which the patient needs support/attention? (write down each need on a card).**	*When e.g. 'being in control' is mentioned:*<br>- *What happens when the patient is in control?*<br>- *Can you tell about a situation in which the patient was in control?*<br>- *What does it mean for the patient to be in control?*
**Q5: Can you place these cards in order of priority (which of these needs need to be resolved by a caregiver first of all)?**<br><br>[Take a picture of the needs in order of priority]	- *Explore why the family caregiver chooses this order.*
**Summarise**	

Comparison of problems and needs to 3 months ago

Take the picture with problems/needs discussed in the baseline interview and show it to the family caregiver.

Questions	Probing opportunities
**Q6: If you compare the problems and needs the patient currently encounters to those on the picture, which problems and needs have changed and which remained the same?**	- *Explore these differences and similarities.*
**Summarise**	

Theme 2: relationships and communication with and between caregivers

Relations between the patient and caregivers

Explain that you would like to explore the current contact between the patient /family caregiver and professional caregivers now. Again you will follow the same steps as in the baseline interview. Then you will compare the current situation to that of 3 months ago.

You are going to use the cards with caregivers on it. First place the 'patient' card in the middle.

An EU Framework 7 Programme (FP7/2007-2013) under grant agreement n° 335555

Questions	Probing opportunities
**Step 1: With which caregivers does the patient have more and less frequent contact? (refer to the SNA of the patient)**<br><br>[Let the family caregiver place the caregivers with whom the patient has most frequent contact closest to the 'patient' card and those with whom (s)he has the least frequent contact further away from the 'patient' card]<br><br>[Take a picture of the frequency of contacts in the care network]	
**Step 2: *What* do these caregivers do when the patient has contact with them?**	- *What kind of care or support is provided?*
**Step 3: *Which* caregivers are most important to the patient?**<br><br>[Let the family caregiver place the caregivers which are more important for the patient closer to the patient card and those who are less important further away].<br><br>[Take a picture showing the caregivers that are more and less important for the patient]	*You can think of importance in two dimensions:*<br><br>- *Relational dimension e.g. caregiver shows acknowledgement (has emphatic attitude); shows commitment; shows involvement*<br>- *Giving dimension e.g. caregiver gives medication/ treatment; gives information (by being clear/honest about the patient's current position and/or prognosis); gives hope*
**Step 4: *Why* are these caregivers important to the patient?**	

An EU Framework 7 Programme (FP7/2007-2013) under grant agreement n° 335555

**Step 5: For which of the 3 highest priority problems and needs (Q3 and Q5) support is currently provided by these caregivers?**<br><br>[Place the card with the problems/needs on the particular caregiver. If there are more caregivers that treat one problem, make more cards with the same problem on it].	
**Step 6: Would you like to mention one additional priority problem/need for which the patient currently receives support, that you did not mention in step 5?**<br><br>[Take picture showing the 3 (or 4) highest priority problems/needs that are currently supported by caregivers and the problems/needs that remain]	- *Explore why this additional problem/need is mentioned.*
**Summarise**	

Relations between the family caregiver and professional caregivers

Now place the card with family caregiver on it on the patient card.

Questions	Probing opportunities
**Repeat steps 1, 2, 3, 4 in order to examine how the family caregiver experiences his/her own contact with caregivers in the patient's network and to discover if there are differences in contact the patient and family caregiver have with caregivers.**<br><br>[Take a picture of the contacts of the contacts between the family caregiver and professional caregivers].	- *Explore the differences between the family caregiver's and patient's contact (frequency and or importance) with professional caregivers.*

An EU Framework 7 Programme (FP7/2007-2013) under grant agreement n° 335555

Comparison of relations between patient and caregivers to 3 months ago

Take the pictures with the patient-caregiver and family-professional caregiver network discussed in the baseline interview and show it to the family caregiver.

Questions	Probing opportunities
**Step 7: If you compare the caregivers with whom the patient currently has contact and what they do to those on the picture, what has changed and what remained the same?**	- *Explore these differences and similarities.*
**Repeat step 7 in order to compare the contact of the family caregiver with professional caregivers in the care network to 3 months ago**	- *Explore these differences and similarities.*
**Summarise**	

Collaboration between caregivers

Explain that after discussing the patient's and family caregiver's contacts with several caregivers, you would like to focus on the collaboration between caregivers. Let the family caregiver draw lines between professional caregivers using markers. You will follow the same procedure as in the baseline interview and will subsequently ask for the differences and similarities compared to 3 months ago.

Questions	Probing opportunities
**Q11: Who, do you think, works together in the care network in front of you. How do you notice this?**<br><br>[Take a picture showing collaboration between caregivers in the network]	- *Explore whether the collaboration is focused on care for the patient or on care for the family caregiver.*
**Q12: What is the importance for the patient, that these caregivers work together? Why?**	

An EU Framework 7 Programme (FP7/2007-2013) under grant agreement n° 335555

**Q13: Who, do you think, should work together? Why?**<br><br>[Take a picture showing caregivers in the network that should work together]	- *Explore why they do not work together now according to the family caregiver.*
**Summarise**	

Comparison of collaboration between caregivers to 3 months ago

Take the picture about collaboration between caregivers in the network discussed in the baseline interview and show it to the family caregiver.

Questions	Probing opportunities
**Q6: If you compare the caregivers who do and do not work together to those on the picture, what has changed and what remained the same?**	- *Explore these differences and similarities.*
**Summarise**	
**Is there something you would like to say or add?**<br><br>**Do you have any questions?**	

Closing

Thank the family caregiver for his/her time.

Explain what will happen from now on and how the patient can come to know more about the results (The results will be available at the end of 2015/early 2016. In the meantime patient can visit website www.insup-c.eu.

➔ **Stop audiotape**

An EU Framework 7 Programme (FP7/2007-2013) under grant agreement n° 335555

Interview protocol: Final interview family caregiver

What to bring
- audio-recorder + extra batteries<br>- note book<br>- pen/pencil

Introduction

1. Building rapport after the first interview + practical matters

- Offer condolences, if not yet done. Ask the family caregiver how (s)he is (building rapport).
- Mention that whenever the family caregiver is not feeling well (s)he can decide to stop the interview at any moment.
- Mention that that are no (right or) wrong answers.

2. What is the aim of this final interview?

To get insight in the bereaved family caregiver's experiences with care provision and bereavement support from the moment that the death of the patient was near until shortly after bereavement.

An EU Framework 7 Programme (FP7/2007-2013) under grant agreement n° 335555

➔ **Start audiotape**

The interview

Questions	
1.	When did you of hear or did it became obvious that the death of your relative was very near? (Who told you?)
2.	After this moment, were there major changes in treatment policy and/or care provision? (Think of e.g. medication stop, treatment stop, sedation, use of LCP, other end-of-life decisions)
3.	What were the most important problems and needs in this phase?<br>(Pay attention to the problems of both the patient and bereaved family caregiver)<br>(Here you can also bring forward whether the family caregiver needed to make decisions on behalf of the patient)
4.	Did you receive care/support to meet these problems/needs? If yes, what care/support did you receive?<br>By which caregivers?<br>What did they do?<br>Whom of these caregivers was most important? Why?
5.	How did you experience care provision by these caregivers in this last phase?<br>Did it meet your problems/needs/expectations?<br>Was care provided with dignity?
6.	Did your relative die at the preferred place?<br>Has (s)he died peacefully?
7.	Which caregivers were involved after the death of your relative?
8.	Remaining questions...

Closing

Thank the family caregiver for his/her time.

Explain what will happen from now on and how the patient can come to know more about the results (The results will be available at the end of 2015/early 2016. In the meantime patient can visit website www.insup-c.eu.

➔ **Stop audiotape**

An EU Framework 7 Programme (FP7/2007-2013) under grant agreement n° 335555

A group interview with professionals – InSup-C

Who are we going to invite?

- For discussion: various healthcare professionals working in/associated to one particular initiative (inclusion at least partly motivated by patient interviews and SNA's)

Physical requirements for conducting the interview

- You will need a space that facilitates undisturbed and calm conversations
- The room has to be big enough for +/- 8 people. You arrange the room so as to stimulate interaction (circle; rectangle)
- The room should have a pleasant climate for 90 minutes
- Think about conducting the interview with an independent conversation leader (if available) and one researcher. At least, make sure that the researcher has time/opportunities to bring in his/her particular knowledge about the initiatives.
- What to bring? White board/flip board; markers; audio recorder suited to record group conversations, need a separate, multi directional microphone

---

This interview guide assumes a 90 minute group session

Preparation

- Consider using PowerPoint – or some other visual method – to display the main questions in each of the steps as prompts for the discussion
- Ask participants to complete the demographic sheet in as much detail as they wish

To start: the formal part

- To give everybody a warm welcome
- Explain the aim of the study
- Explain the aim of the group interview:
  "in this interview we would like to explore how you experience, or have experienced working with this/being part of this integrated palliative care initiative .... All of you have somehow been involved in patient care through this initiative, and we would like you to

An EU Framework 7 Programme (FP7/2007-2013) under grant agreement n° 335555

discuss how the multidisciplinary collaboration contributed or, perhaps, impeded high-quality care for the patient. In more general terms: “How is the initiative doing so far?”

- We are especially interested in what goes well in delivering integrated palliative care in the initiative. What are the things they are proud of? Why does that succeed; what can others learn from that?

- We will pose initial questions and/or present propositions to start up the discussion. Please feel free to share your opinions and feelings or to respond to one of the other members of this group. The conversations will be audio-recorded and transcribed. Both the audio files and the transcripts will be kept confidential. Only the researchers have access to the research material. All quotes used in research papers or presentations will be anonymised.

<u>**Ground rules**</u>

It is useful to have these ground rules on a slide or on flip chart paper for you (and they) to refer to

- Ask participants to switch off phones (or switch to silent if they need to remain available)
- Suggest that if participants need to leave the room (or leave the session) that they do so quietly
- Ask participants to respect confidentiality i.e. not to repeat or talk about what is said in the group interview to others (this is important as many of the people present will continue to work together and it ‘frees up’ people to be more honest in the discussion)
- Ask participants to treat each other’s comments and opinions with respect. It is possible to disagree – but to do so respectfully
- Remind people that the conversation will be recorded and transcribed, so it is important not to speak over each other or at the same time. Tell them that you may remind them of this again if the discussion gets enthusiastic!
- **Before starting the interview, please make an introduction round.**

An EU Framework 7 Programme (FP7/2007-2013) under grant agreement n° 335555

The logic behind the interview

To prepare for the group interview, we suggest that you and the independent chair/conversation leader (re-)read the presentation of Jeroen van Wijngaarden, January 20, 2015, and the article 'Understanding integrated care: a comprehensive conceptual framework based on the integrative functions of primary care' by Valentijn et al. to (re)gain a feel for the dimensions of integrated care. You will need this knowledge of integrated care to check, during the interview, whether participants' answers cover all important dimensions of integrated care. In other words, this knowledge will help you to ask critical probing questions in case of one-dimensional answers.

Also look at the interview material with the patients. Try to identify what they particularly appreciate in this initiative. Present these examples during the group interview to try to understand what is required to get these results.

The interview process

We will invite the group to 'think out loud' about the questions and statements. If a second researcher is present (or a researcher next to the conversation leader), he/she can write key words/phrases on the whiteboard/flipchart so that these may be referred to during the interview: to note links and connections and/or to explore aspects that may have received scant attention.

(While participants are discussing the answers, please stay alert to pick up cues with which you can further the discussion)

Step 1: Description of the integrated palliative care initiative – the patients

a. Please describe a situation in which you believe a patient received good quality integrated palliative care. Why did that go so well?
b. What are in general the things that you are proud of in your initiative? Why does that work so well and what can others learn from that?
c. Please describe a situation in which you believe a patient received poor quality integrated palliative care. Why did that happen and what can we learn from that?
d. What makes the difference between good and bad integrated palliative care?
e. What are the particular 'types' or characteristics of patients for whom integrated palliative

care works best/worst?

f. What difference – if any – does the particular condition (cancer, COPD, CHF) make?

g. Ideally, at what moment is a patient referred to/admitted into the integrated palliative care initiative?

h. Which professionals are involved with patients at what time(s)?

Try to stimulate discussion as much as possible: ask clarifying questions; ask participants to respond to one another; ask if participants experiences are similar or different. Mentioned below are a few propositions that ***could*** *help stimulate the discussion as well (these propositions contain elements that are available for probing. E.g., the 'how-question'):*

1. Please respond: "Well integrated palliative care covers appropriate assessment at first appointment and on-going review during the disease trajectory."
2. Please respond: "Well integrated palliative care guarantees timely access to services based on patient/family needs and wishes."
3. Please respond: "Well integrated palliative care supports choice and personalized care using tools (where appropriate) such as: advance care plans; advance decision to refuse treatment; do not attempt cardio-pulmonary resuscitation (DNAR) orders; preferred priorities of care etc."

Step 2: Description of the integrated palliative care initiative – communication and information

In this second step, we focus on communication and the sharing of information. Let participants react to the following questions:

1. How does the initiative provide the right information, at the right time?
   a. For patients?
   b. For family caregivers?
   c. For professionals?
   d. What is "the right information, at the right time" (at least)?
   e. How do information needs differ – if at all – between the conditions this study is concerned with (cancer, COPD, CHF)?
2. What communication channels are used by professionals?
3. What are some of the enablers and barriers to good communication?

a. Between professionals and patients/carers?
    i. How are patients included in care planning and decision making?
    ii. How are family caregivers included in care planning and decision making?
b. Between professionals?
    i. How are changes to individual care plans made and communicated to all members of the wider care team
c. Between agencies in the initiative?
d. With other agencies not connected to the initiative **(mentioned in the patient/carer interviews)**?

Mentioned below are a few propositions that ***could*** *help stimulate the discussion as well (these propositions contain elements that are available for probing. E.g., the 'how-question'):*

1. Please respond: "Well integrated palliative care facilitates regular and open conversations about end of life needs, patient values, and patient preferences as well as advanced care planning." + Whether and how should this knowledge be shared with other caregivers.
2. Please respond: "Well integrated palliative care produces information/facilitates appointments about treatment and care during the disease trajectory and towards the end of life – including where the person wishes to die and what services are available at that time and for bereaved people".

You could offer a BREAK to participants if appropriate.

Step 3: Description of the integrated palliative care initiative – the content of care

In this third step, we will focus on the content and continuity of care. Let participants react to the following questions.

a. What makes or who ensures that the patient receives the right level of palliative care at the right moment in the disease trajectory?
b. How is the continuity of care attended to and delivered within the initiative/across care givers and organisations?

An EU Framework 7 Programme (FP7/2007-2013) under grant agreement n° 335555

c. How is palliative care delivery aligned with the patients' and family caregivers' wishes?
d. How is 'holistic patient assessment' assured? I.e. that all aspects of care are covered: physical, psychological, spiritual and social?
e. What are the local provisions for care overnight and at weekends?

*Below, you'll find a few propositions that **could** help stimulate the discussion as well (these propositions contain elements that are available for probing. E.g., the 'how-question'):*

1. Please respond: "Well integrated palliative care facilitates open communication about death and dying based on patients and family needs."
2. Please respond: "Well integrated palliative care contains evaluation of pain and other physical and psychological symptoms with adequate access to medications and equipment."
3. Please respond: "Well integrated palliative care contains multidisciplinary care services at generalist and specialist level to provide high quality care at any time during day or night, based on the patient's condition, care plan and wishes."
4. Please respond: "Well integrated palliative care attends to the support needs of bereaved carers".

Step 4: Description of the integrated palliative care initiative – availability of materials and personnel

1. How does 'the initiative' ensure that people with expert knowledge are available to supply the right medication/equipment at the right time?
2. How is the provision of drugs – particularly pain relief and drugs for use at end of life – organised and who takes responsibility for this? **(issues about medication that are raised in the patient/carer interviews)**
3. How is the provision of ADL aids and other equipment organised and who takes responsibility for this?

In addition to the questions/propositions: you could prepare yourself by reading through the interview material and use some striking elements concerning [e.g., patient logistics] to challenge/compliment participants and to further stimulate discussion. If the interview is chaired

by another person than the researcher, you'll have to provide the chair beforehand with these 'striking elements'.

Closure

* Thank the participants for partaking in this focus group. Explain them what will happen from here on with the research material and when and how they will be able to read anything about the research project. If suitable in your country, hand over to the participants the small present for participation and inform them about reimbursement of travel expenses.

Refreshments

Suggestion: read the information below for additional information on what to expect. Some elements could help you to build probing questions or pick critical elements from the interviews. This information also gives you a first insight into the elements we will be looking for in the analysis.

- **Delivery system integration:** the extent at which at the macro level of the care system financing and regulation are aligned for palliative care within and between cure, care and social services. Hindering and stimulating factors at this level can be identified for example:
  - Information logistic may be problematic if regulations do not allow to share patient information across hospital and social care.
  - Integration may be stimulated if cure, care and social services are allowed to transfer and share resources.

- **Functional integration:** The extent at which at the meso level support activities (finance, management and information systems) and organizational structures are aligned for palliative care within and between cure, care and social services.
  What are the structures they introduced at this level or what are the hindering factors; for example:
  - sharing administration to avoid duplication of activities.
  - Introducing an electronic medical record across settings
  - Building an organisation that works across cure, care and social care.

An EU Framework 7 Programme (FP7/2007-2013) under grant agreement n° 335555

- **Clinical integration:** The extent at which at the micro level care delivery activities are aligned between care givers for palliative care within and between cure, care and social services. What has helped integration at this level and what are hindrances;
    - sharing patient information; formal (paper; electronic patient files?) & informal (telephone/teleconsultation/e-mail)
    - multidisciplinary team meetings for decision making (*let participants explain the choices that have been made; let them explain the (potential) value of multidisciplinary team meetings*)
    - patient/family caregiver-inclusive team meetings for decision making (ibid.)
    - collaborative interventions at a patient’s bedside (ibid.)
    - *protocols/pathways*
    - (therapeutic) continuity for the patient
    - attuned care for the patient
    - collaboration with the patient/family caregiver

- **Cultural integration**: The extent at which norms, values and approaches of care givers are aligned.
  What helped them align norms and values or where do they experience problems.
    - training activities and evaluations; within and between disciplines/institutions (ibid.)
    - a shared philosophy on paper; a signed mission statement. (What did your initiative do to co-create such a philosophy?)
    - collaboration towards a shared philosophy in practice. (What has been done to implement the philosophy into daily practice? How did that work out?)

- **Social integration:** The extent at which social relations between caregivers are intensified; trust.
  What did they do to intensify relationships and trust, where do they experience problems?
    - visits; internships, meetings.
    - concrete definitions of the different responsibilities and roles of the various professionals/volunteers/patients/family caregivers
    - versatile and flexible professionals who can build on each other

- **Strategic integration:** The extent at which goals, means, power and interests of organizations and caregivers are aligned.
  What did they do to align goals, means, power and interests. Where do they experience problems?

An EU Framework 7 Programme (FP7/2007-2013) under grant agreement n° 335555

- Hired an independent project coordinator
- Used a specific implementation strategy.
