# Supplementary material for: The experiences of patients with advanced heart failure, family carers, and health professionals with palliative care services: a secondary reflexive thematic analysis of longitudinal interview data
Source: BMC Palliat Care. 2023 Aug 10;22:115. doi: 10.1186/s12904-023-01241-1 (PMC10413510; doi:10.1186/s12904-023-01241-1)
Supplement: Supplementary file 2 — Supplementary Material 2 [file 12904_2023_1241_MOESM2_ESM.docx]

Additional file 2: Braun and Clarke’s checklist of criteria for good thematic analysis

| Process | Quality criteria | Notes |
| --- | --- | --- |
| Transcription | Transcribing the data to an appropriate level of detail and checking the transcripts against the tapes for accuracy. | - This was not applicable as the secondary analyst (BR) did not transcribe the interviews nor did he have access to tapes. However, all interviews were transcribed verbatim by a transcription company and checked by experienced, well-trained researchers. |
|  | | |
| Coding | Giving equal attention to each data item in coding. | - BR coded all the interviews systematically. The health professional group interviews were coded under codes created from the patient and family interviews to compare their opinions. However, where data in the group interviews could not be associated with existing codes, new codes were created. |
|  | Thorough, inclusive, and comprehensive coding (themes not generated from a few vivid examples). | - BR combined coding small chunks of data to open up the data and not miss important meanings and nuances in the text (mainly for the first few interviews) with coding longer segments of data to investigate broad and overarching discussion areas and not lose the context. - BR checked the data under each code to explore both exemplars and outliers that oppose the theme’s general perspective. - BR created a tally mark chart for each theme to ensure that data extracts from different participants were provided to support the themes’ prevalence in the data. |
|  | Collating all relevant extracts for each theme. | - BR used computer software to facilitate managing data and collating coded extracts for each theme. |
|  | Checking the themes against each other and the original dataset. | - BR compared the themes with the coded extracts and interview transcripts to confirm their presence in the data. |
|  | Internally coherent, consistent, and distinctive themes. | - BR compared the themes against each other to evaluate if each has a distinctive central organising concept (core idea) and little overlap. |
|  | | |
| Analysis | Analysing rather than just paraphrasing or describing the data. | - BR combined semantic and latent approaches to generate the themes. In both cases, the analysis went beyond the data content to explore meanings. |
|  | Matching between data extracts and analytic claims. | - BR chose data extracts that best illustrate the themes. |
|  | Telling a well-organised story about the data and topic. | - Data extracts were embedded within an analytic narrative to tell a coherent story. - BR created a thematic map that shows how themes relate to each other to tell a convincing story about the data and answer the research question. |
|  | Good balance between data extracts and analytic narrative. | - BR supported each theme with a range of data extracts. |
|  | | |
| Overall | Allocating enough time to complete all analysis phases. | - It took BR about one year to complete the analysis which provided an in-depth understanding of the data. |
|  | | |
| Written Report | Explicating the assumptions about and approach to thematic analysis. | - BR discussed his assumptions and illustrated the rationale of his analysis approach (mainly inductive and semantic). |
|  | Consistency between the described method and reported analysis. | - BR checked the final report against the adopted method and epistemology to make sure that he has done what he claimed to do. |
|  | Consistency between the report language and concepts and the analysis epistemological position. | - BR explained how reflexive thematic analysis is concordant with his philosophical and theoretical assumptions (pragmatism and Normalisation Process Theory), which were reflected in the report language. |
|  | Positioning the researcher as active in the research process. | - Throughout the analysis process, BR reflected on how his assumptions, past experiences, and possible biases shaped his interpretation of the results. Consequently, themes were developed, rather than emerged, from the data. |

Description of data: A comprehensive check of the quality of the thematic analysis based on Braun and Clarke’s Checklist of Criteria for Good Thematic Analysis

File format: .docx
