# Supplementary material for: The experiences of patients with advanced heart failure, family carers, and health professionals with palliative care services: a secondary reflexive thematic analysis of longitudinal interview data
Source: BMC Palliat Care. 2023 Aug 10;22:115. doi: 10.1186/s12904-023-01241-1 (PMC10413510; doi:10.1186/s12904-023-01241-1)
Supplement: Supplementary file 3 — Supplementary Material 3 [file 12904_2023_1241_MOESM3_ESM.docx]

Additional file 3: Criteria for determining the general quality of the primary study dataset

| Criteria | Options | | |
| --- | --- | --- | --- |
| Ready access to study documents and team | **Yes** | | **No** |
| Tapes of interviews | -- | | **✓** |
| Hard copies/transcripts of interviews | **✓** | | -- |
| Field notes | -- | | **✓** |
| Memos or interpretive notes | Not relevant as the secondary analyst (BR) did his own analysis | | |
| Principal investigator or team members | **✓** | | -- |
| Training of primary team | **Satisfactory** | **Unable to determine** | **Unsatisfactory** |
| Credentials of team members to conduct the primary study | **✓** | **--** | **--** |
| Training of members for roles in the primary study | **✓** | **--** | **--** |
| Completeness of dataset | **Yes** | | **No** |
| Available documents are complete | **✓** | | **--** |
| Accuracy of transcription | Cannot assess | | |
| Minimal or insignificant typographic errors | **✓** | | **--** |
| Appropriate use of software | Not relevant as BR did his own analysis | | |
| Ability to assess interviewing quality | **Satisfactory** | **Unable to determine** | **Unsatisfactory** |
| Interviewing quality | **--** | **✓** | **--** |
| Interviewing format allowed responses of descriptive depth | **✓** | **--** | **--** |
| Focus, meaning, and subject of responses can be determined | **✓** | **--** | **--** |
| Ability to assess sampling plan | **Yes** | | **No** |
| Type of sampling plan (for example, convenience, purposive, theoretical) is clear | **✓** | | **--** |
| Criteria for determining the fit of the secondary research question | **Present in sufficient depth** | **Unable to determine** | **Not present in sufficient depth** |
| Ability to determine the extent to which the concept of interest is reflected in the dataset | **✓** | **--** | **--** |
| Ability to estimate the validity of the new question | **Likely** | **Unsure** | **Unlikely** |
| Study sample could be expected to experience this concept or situation | **✓** | -- | -- |
|  | **Similar** | **Somewhat similar** | **Dissimilar** |
| Proposed research question is similar to that in the primary study | **✓** | **--** | **--** |
| Aggregate impression | **Yes** | | **No** |
| Dataset of sufficient quality, completeness, and fit with the secondary research question | **✓** | | **--** |

Description of data: A comprehensive check of the quality of the primary study dataset for secondary data analysis

File format: .docx
